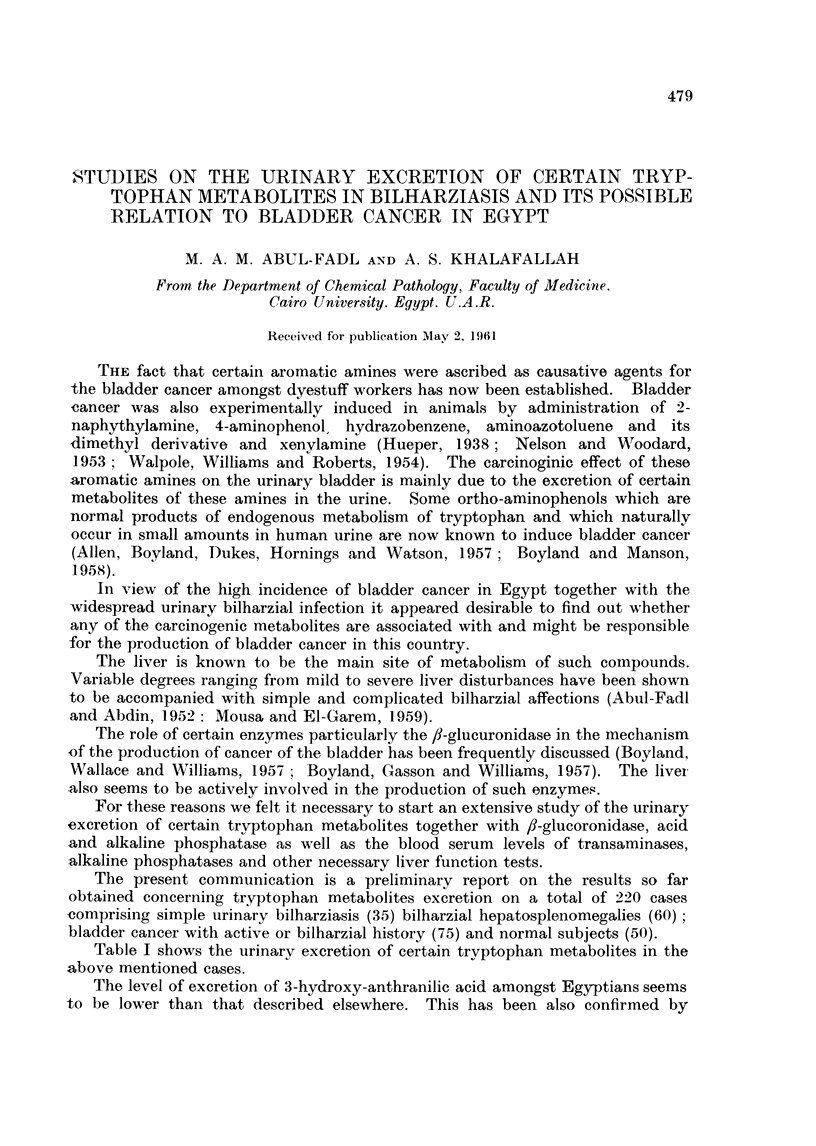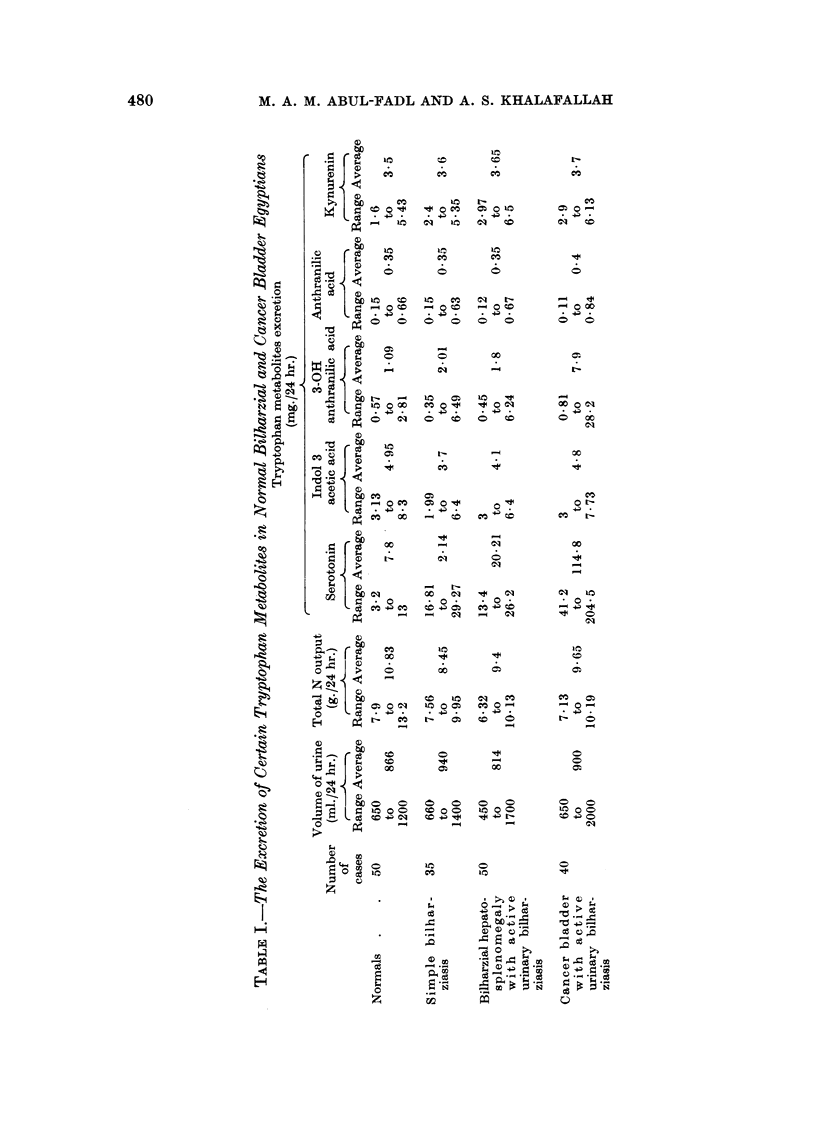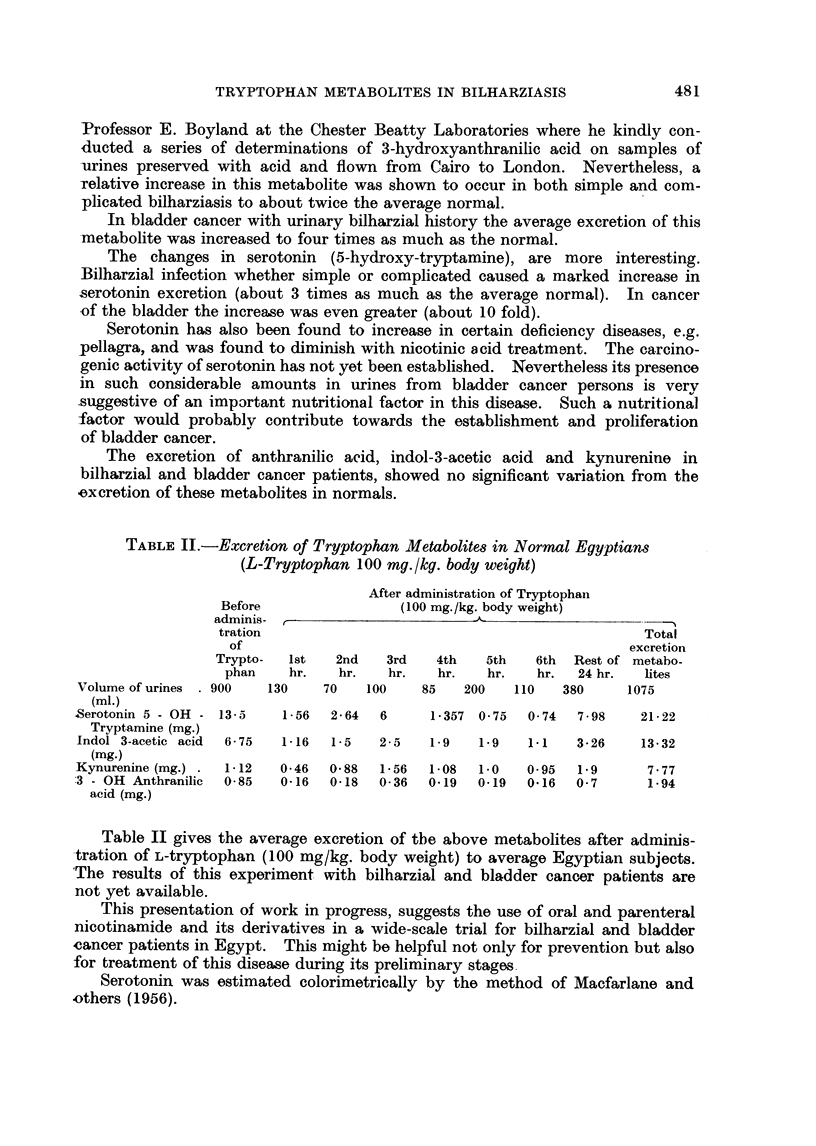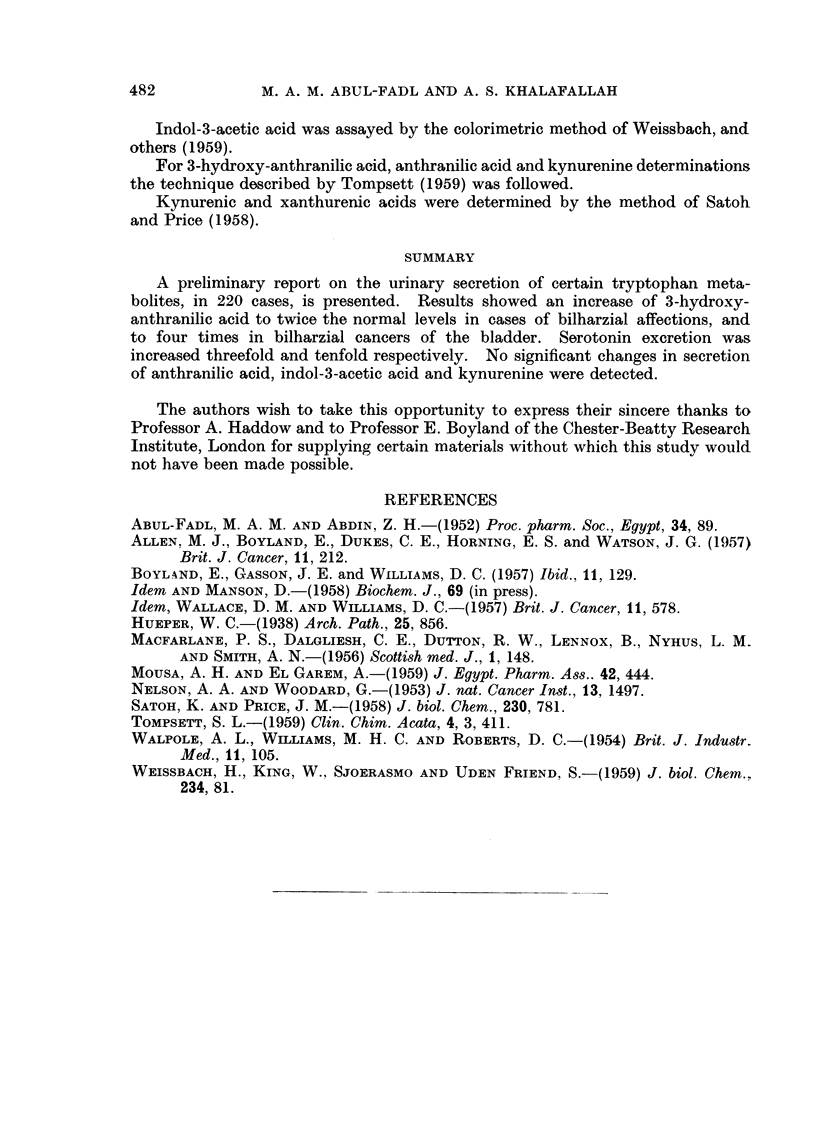# Studies on the Urinary Excretion of Certain Tryptophan Metabolites in Bilharziasis and its Possible Relation to Bladder Cancer in Egypt

**DOI:** 10.1038/bjc.1961.57

**Published:** 1961-09

**Authors:** M. A. M. Abul-Fadl, A. S. Khalafallah


### STUDIES ON THE URINARY EXCRETION OF CERTAIN TRYPTOPHAN METABOLITES IN BILHARZIASIS AND ITS POSSIBLE RELATION TO BLADDER CANCER IN EGYPT

M. A. M. ABUL-FADL AND A. S. KHALAFALLAH

*From the Department of Chemical Pathology, Faculty of Medicine, Cairo University, Egypt, U.A.R.*

Received for publication May 2, 1961

THE fact that certain aromatic amines were ascribed as causative agents for the bladder cancer amongst dyestuff workers has now been established. Bladder cancer was also experimentally induced in animals by administration of 2-naphythylamine, 4-aminophenol, hydrazobenzene, aminoazotoluene and its dimethyl derivative and xenylamine (Hueper, 1938; Nelson and Woodard, 1953; Walpole, Williams and Roberts, 1954). The carcinoginic effect of these aromatic amines on the urinary bladder is mainly due to the excretion of certain metabolites of these amines in the urine. Some ortho-aminophenols which are normal products of endogenous metabolism of tryptophan and which naturally occur in small amounts in human urine are now known to induce bladder cancer (Allen, Boyland, Dukes, Hornings and Watson, 1957; Boyland and Manson, 1958).

In view of the high incidence of bladder cancer in Egypt together with the widespread urinary bilharzial infection it appeared desirable to find out whether any of the carcinogenic metabolites are associated with and might be responsible for the production of bladder cancer in this country.

The liver is known to be the main site of metabolism of such compounds. Variable degrees ranging from mild to severe liver disturbances have been shown to be accompanied with simple and complicated bilharzial affections (Abul-Fadl and Abdin, 1952; Mousa and El-Garem, 1959).

The role of certain enzymes particularly the $\beta$-glucuronidase in the mechanism of the production of cancer of the bladder has been frequently discussed (Boyland, Wallace and Williams, 1957; Boyland, Gasson and Williams, 1957). The liver also seems to be actively involved in the production of such enzymes.

For these reasons we felt it necessary to start an extensive study of the urinary excretion of certain tryptophan metabolites together with $\beta$-glucoronidase, acid and alkaline phosphatase as well as the blood serum levels of transaminases, alkaline phosphatases and other necessary liver function tests.

The present communication is a preliminary report on the results so far obtained concerning tryptophan metabolites excretion on a total of 220 cases comprising simple urinary bilharziasis (35) bilharzial hepatosplenomegalies (60); bladder cancer with active or bilharzial history (75) and normal subjects (50).

Table I shows the urinary excretion of certain tryptophan metabolites in the above mentioned cases.

The level of excretion of 3-hydroxy-anthranilic acid amongst Egyptians seems to be lower than that described elsewhere. This has been also confirmed by

TABLE I.—*The Excretion of Certain Tryptophan Metabolites in Normal Bilharzial and Cancer Bladder Egyptians*

| | Number of cases | Volume of urine (ml./24 hr.) | | Total N output (g./24 hr.) | | Tryptophan metabolites excretion (mg./24 hr.) Serotonin | | Indol 3 acetic acid | | 3-OH anthranilic acid | | Anthranilic acid | | Kynurenin | |
|---|---|---|---|---|---|---|---|---|---|---|---|---|---|---|---|
| | | Range | Average | Range | Average | Range | Average | Range | Average | Range | Average | Range | Average | Range | Average |
| Normals | 50 | 650 to 1200 | 866 | 7·9 to 13·2 | 10·83 | 3·2 to 13 | 7·8 | 3·13 to 8·3 | 4·95 | 0·57 to 2·81 | 1·09 | 0·15 to 0·66 | 0·35 | 1·6 to 5·43 | 3·5 |
| Simple bilharziasis | 35 | 660 to 1400 | 940 | 7·56 to 9·95 | 8·45 | 16·81 to 29·27 | 2·14 | 1·99 to 6·4 | 3·7 | 0·35 to 6·49 | 2·01 | 0·15 to 0·63 | 0·35 | 2·4 to 5·35 | 3·6 |
| Bilharzial hepato-splenomegaly with active urinary bilharziasis | 50 | 450 to 1700 | 814 | 6·32 to 10·13 | 9·4 | 13·4 to 26·2 | 20·21 | 3 to 6·4 | 4·1 | 0·45 to 6·24 | 1·8 | 0·12 to 0·67 | 0·35 | 2·97 to 6·5 | 3·65 |
| Cancer bladder with active urinary bilharziasis | 40 | 650 to 2000 | 900 | 7·13 to 10·19 | 9·65 | 41·2 to 204·5 | 114·8 | 3 to 7·73 | 4·8 | 0·81 to 28·2 | 7·9 | 0·11 to 0·84 | 0·4 | 2·9 to 6·13 | 3·7 |

Professor E. Boyland at the Chester Beatty Laboratories where he kindly conducted a series of determinations of 3-hydroxyanthranilic acid on samples of urines preserved with acid and flown from Cairo to London. Nevertheless, a relative increase in this metabolite was shown to occur in both simple and complicated bilharziasis to about twice the average normal.

In bladder cancer with urinary bilharzial history the average excretion of this metabolite was increased to four times as much as the normal.

The changes in serotonin (5-hydroxy-tryptamine), are more interesting. Bilharzial infection whether simple or complicated caused a marked increase in serotonin excretion (about 3 times as much as the average normal). In cancer of the bladder the increase was even greater (about 10 fold).

Serotonin has also been found to increase in certain deficiency diseases, e.g. pellagra, and was found to diminish with nicotinic acid treatment. The carcinogenic activity of serotonin has not yet been established. Nevertheless its presence in such considerable amounts in urines from bladder cancer persons is very suggestive of an important nutritional factor in this disease. Such a nutritional factor would probably contribute towards the establishment and proliferation of bladder cancer.

The excretion of anthranilic acid, indol-3-acetic acid and kynurenine in bilharzial and bladder cancer patients, showed no significant variation from the excretion of these metabolites in normals.

TABLE II.—*Excretion of Tryptophan Metabolites in Normal Egyptians (L-Tryptophan 100 mg./kg. body weight)*

| | Before adminis-tration of Trypto-phan | After administration of Tryptophan (100 mg./kg. body weight) | | | | | | | |
|---|---|---|---|---|---|---|---|---|---|
| | | 1st hr. | 2nd hr. | 3rd hr. | 4th hr. | 5th hr. | 6th hr. | Rest of 24 hr. | Total excretion metabo-lites |
| Volume of urines (ml.) | 900 | 130 | 70 | 100 | 85 | 200 | 110 | 380 | 1075 |
| Serotonin 5 - OH - Tryptamine (mg.) | 13·5 | 1·56 | 2·64 | 6 | 1·357 | 0·75 | 0·74 | 7·98 | 21·22 |
| Indol 3-acetic acid (mg.) | 6·75 | 1·16 | 1·5 | 2·5 | 1·9 | 1·9 | 1·1 | 3·26 | 13·32 |
| Kynurenine (mg.) | 1·12 | 0·46 | 0·88 | 1·56 | 1·08 | 1·0 | 0·95 | 1·9 | 7·77 |
| 3 - OH Anthranilic acid (mg.) | 0·85 | 0·16 | 0·18 | 0·36 | 0·19 | 0·19 | 0·16 | 0·7 | 1·94 |

Table II gives the average excretion of the above metabolites after administration of L-tryptophan (100 mg/kg. body weight) to average Egyptian subjects. The results of this experiment with bilharzial and bladder cancer patients are not yet available.

This presentation of work in progress, suggests the use of oral and parenteral nicotinamide and its derivatives in a wide-scale trial for bilharzial and bladder cancer patients in Egypt. This might be helpful not only for prevention but also for treatment of this disease during its preliminary stages.

Serotonin was estimated colorimetrically by the method of Macfarlane and others (1956).

Indol-3-acetic acid was assayed by the colorimetric method of Weissbach, and others (1959).

For 3-hydroxy-anthranilic acid, anthranilic acid and kynurenine determinations the technique described by Tompsett (1959) was followed.

Kynurenic and xanthurenic acids were determined by the method of Satoh and Price (1958).

SUMMARY

A preliminary report on the urinary secretion of certain tryptophan metabolites, in 220 cases, is presented. Results showed an increase of 3-hydroxy-anthranilic acid to twice the normal levels in cases of bilharzial affections, and to four times in bilharzial cancers of the bladder. Serotonin excretion was increased threefold and tenfold respectively. No significant changes in secretion of anthranilic acid, indol-3-acetic acid and kynurenine were detected.

The authors wish to take this opportunity to express their sincere thanks to Professor A. Haddow and to Professor E. Boyland of the Chester-Beatty Research Institute, London for supplying certain materials without which this study would not have been made possible.

### REFERENCES

Abul-Fadl, M. A. M. and Abdin, Z. H.—(1952) *Proc. pharm. Soc., Egypt*, **34**, 89.

Allen, M. J., Boyland, E., Dukes, C. E., Horning, E. S. and Watson, J. G. (1957) *Brit. J. Cancer*, **11**, 212.

Boyland, E., Gasson, J. E. and Williams, D. C. (1957) *Ibid.*, **11**, 129.

*Idem* and Manson, D.—(1958) *Biochem. J.*, **69** (in press).

*Idem*, Wallace, D. M. and Williams, D. C.—(1957) *Brit. J. Cancer*, **11**, 578.

Hueper, W. C.—(1938) *Arch. Path.*, **25**, 856.

Macfarlane, P. S., Dalgliesh, C. E., Dutton, R. W., Lennox, B., Nyhus, L. M. and Smith, A. N.—(1956) *Scottish med. J.*, **1**, 148.

Mousa, A. H. and El Garem, A.—(1959) *J. Egypt. Pharm. Ass..* **42**, 444.

Nelson, A. A. and Woodard, G.—(1953) *J. nat. Cancer Inst.*, **13**, 1497.

Satoh, K. and Price, J. M.—(1958) *J. biol. Chem.*, **230**, 781.

Tompsett, S. L.—(1959) *Clin. Chim. Acata*, **4**, 3, 411.

Walpole, A. L., Williams, M. H. C. and Roberts, D. C.—(1954) *Brit. J. Industr. Med.*, **11**, 105.

Weissbach, H., King, W., Sjoerasmo and Uden Friend, S.—(1959) *J. biol. Chem.*, **234**, 81.